# Development of a Pericapsular Elbow Desensitization Technique in Dogs—A Canine Cadaveric Study

**DOI:** 10.3390/vetsci12040374

**Published:** 2025-04-17

**Authors:** Diego A. Portela, Raiane A. Moura, Mariana Cavalcanti, Penny J. Regier, Marta Romano, Adam W. Stern, Enzo Vettorato, Pablo E. Otero

**Affiliations:** 1 Department of Comparative, Diagnostic and Population Medicine, College of Veterinary Medicine, University of Florida, Gainesville, FL 32608, USA; mouraraiane@ufl.edu (R.A.M.); mcaval@midwestern.edu (M.C.); marta.romano@ufl.edu (M.R.); adamstern@ufl.edu (A.W.S.); evettorato@ufl.edu (E.V.); 2 Department of Small Animal Clinical Sciences, College of Veterinary Medicine, University of Florida, Gainesville, FL 32608, USA; pennyjregier@gmail.com; 3 Department of Anesthesiology and Pain Management, Facultad de Ciencias Veterinarias, Universidad de Buenos Aires, Buenos Aires 1427, Argentina; potero@fvet.uba.ar

**Keywords:** canine, innervation, joint, osteoarthritis, regional anesthesia, ultrasound, interventional pain medicine

## Abstract

Peripheral nerve blocks play a critical role in pain management; however, no technique has been described to specifically target the articular nerve branches of the elbow in dogs. This study aimed to develop and compare the success rate of an ultrasound-guided versus a blind pericapsular elbow desensitization (PED) technique in canine cadavers. Phase I involved gross and ultrasonographic evaluations of the elbow joint in four cadavers to identify landmarks for the PED technique. Phase II compared the ultrasound-guided and blind PED techniques using dye injections in eight cadavers, followed by the dissection and histological analysis of stained tissues to confirm nerve involvement. The results demonstrated that four quadrant injections are required to target the articular branches of the radial, ulnar, median, and musculocutaneous nerves. The ultrasound-guided technique achieved a significantly higher overall success rate (77.5%) than the blind technique (45%, *p* = 0.005). While both techniques showed similar success rates (50–62.5%) for the ulnar, median, and musculocutaneous branches, the ultrasound-guided technique (100%) outperformed the blind technique for the cranial (37%, *p* = 0.02) and caudal (12.5%, *p* = 0.001) articular branches of the radial nerve. These findings provide a foundation for future clinical studies evaluating the ultrasound-guided PED technique in live animals.

## Simple Summary

Common methods for managing osteoarthritic elbow pain in small animals often fail to provide long-term relief, negatively impacting the animal’s quality of life. Peripheral nerve blocks, such as brachial plexus blocks, can offer regional analgesia for the elbow but frequently result in impaired limb motor function. This study aimed to develop and compare two approaches—ultrasound-guided and blind injection techniques—to selectively target the articular nerve branches responsible for elbow joint sensory innervation in canine cadavers. The research introduced the pericapsular elbow desensitization (PED) technique, demonstrating that the ultrasound-guided method achieved significantly higher success rates in affecting the targeted articular branches than the blind technique. These findings establish the foundational groundwork for the PED technique and provide a basis for future clinical studies to evaluate its effectiveness in live animals.

## 1. Introduction

Osteoarthritis (OA) is a chronic, degenerative condition affecting 20% of dogs older than one year and up to 80% of dogs over eight years [1]. It primarily affects highly movable joints, with the highest incidence in the elbow, stifle, and hip [2]. The multimodal management of OA includes weight control, dietary modification, supplements, physical therapy, and non-steroidal anti-inflammatory drugs (NSAIDs) [3]. However, common pain management strategies often fail to provide adequate relief in advanced OA, and chronic NSAID use can lead to severe side effects, requiring treatment discontinuation and impairing quality of life, sometimes necessitating euthanasia [4].

Interventional pain medicine uses techniques like nerve blocks, trigger point injections, and radiofrequency ablation to alleviate chronic pain. To extend pain relief, sensory nerves can be desensitized with drug combinations, such as local anesthetics with steroids, dexmedetomidine, or buprenorphine [5]. Notably, some studies have demonstrated pain relief lasting up to six months following perineural injections of local anesthetics combined with steroids targeting capsular joint nerves in dogs [6].

Ultrasonography has emerged as an indispensable modality for accurately identifying injection sites for nerve blocks. While its application in targeting the articular branches of the hip and knee joints in dogs has been documented (e.g., PHD block [7]; PKD block [8]), a technique for desensitizing the articular branches of the radial, ulnar, median, and musculocutaneous nerves associated with the elbow joint remains unexplored.

This present prospective cadaveric study aimed to develop a regional anesthesia technique for selectively targeting the nerves responsible for the sensory innervation of the elbow joint in dogs. Furthermore, it compared the success rates of elbow articular nerve staining using either a blind technique or an ultrasound-guided approach. The hypothesis tested was that ultrasound guidance improves the overall successful pericapsular injections compared to the blind technique.

## 2. Materials and Methods

The study was designed as a prospective, randomized, anatomic, and cadaveric study. It utilized a total of 12 canine cadavers (i.e., 24 thoracic limbs) that had been euthanized or had died due to medical conditions unrelated to the study. The canine cadavers were donated by clients of the Small Animal Veterinary Teaching Hospital, University of Florida, for teaching or research purposes. The University of Florida does not require approved IACUC protocols for research conducted on cadavers of animals euthanized for reasons unrelated to the study. Specimens with visible abnormalities in the thoracic limbs were excluded.

The study was conducted in two phases. Phase I analyzed the gross anatomical features of the nerves responsible for the sensory innervation of the elbow joint capsule using four canine cadavers (i.e., a total of eight thoracic limbs). Phase II was designed to compare the ultrasound-guided versus the blind injection techniques developed in Phase I in eight canine cadavers (i.e., a total of 16 thoracic limbs). All the cadavers, previously frozen, were thawed at room temperature for 48 to 72 h, and the hair over the anatomical areas corresponding to the elbow was clipped.

- Phase I: Sonoanatomy of the elbow and gross anatomical dissection.

The objective of this phase was to study the ultrasonography of the elbow region, examine the gross anatomy of the nerves involved in the sensory innervation of the elbow joint capsule [9], and develop a pericapsular elbow desensitization (PED) technique to target the articular branches of the elbow using both blind and ultrasound-guided methods.

Initially, four thoracic limbs from two thawed medium-sized breed canine cadavers were dissected to expose the mid-humeral region. First, a medial incision was made to expose the brachial fascia and the underlying median, ulnar, and musculocutaneous nerves. Consequently, a lateral incision was made to expose the radial nerve and its branches by removing the lateral head of the triceps muscle. These nerves were traced distally toward the elbow joint capsule to identify their articular branches using a binocular surgical microscope (Storz Urban US-1 surgical microscope; Storz, St. Louis, MO, USA) following the protocol described by Zamprogno et al. [10]. Bony landmarks for identifying articular branches were determined through palpation and ultrasound in the dissected limbs and correlated with surface landmarks.

Next, one small and one medium-sized thawed cadaver were used to explore the sonoanatomy elbow joint. The goal was to establish relationships between ultrasound images and gross anatomical findings and to identify possible landmarks for ultrasound-guided PED injections. Before scanning, the brachial vessels were dissected in the axillary region and infused with ultrasound gel, as described by Hocking and McIntyre, to enhance visualization during imaging [11]. Ultrasound gel was applied to improve acoustic probe coupling. An experienced anesthesiologist conducted the ultrasound study using a portable ultrasound machine (SonoSite Edge; SonoSite Inc., Bothell, WA, USA) and a linear array transducer (15-6 MHz HFL50x or 13-6 L25x; SonoSite Inc.). The most promising needle access routes were identified during this phase.

Finally, 0.3 mL methylene blue trial injections into each target articular nerve were performed using the identified landmarks. Blind injections were performed on one side of each cadaver, while ultrasound-guided injections were administered on the contralateral side. Subsequent dissections of the injected limbs were conducted to assess the accuracy of dye deposition in relation to the target nerves and refine the injection techniques for optimal application.

- Phase II: Comparison of blind versus ultrasound-guided PED techniques

This phase of the study aimed to compare the success rate of staining the articular branches responsible for the innervation of the elbow joint using either a blind or ultrasound-guided technique. The study was designed to test the hypothesis that ultrasound guidance would result in a greater rate of nerve staining than a blind technique after dye solution injection.

The right and left thoracic limbs were randomly assigned using an online random list generator (www.random.org) to one of the following treatments: (1) ultrasound-guided injections (Group US), where the articular nerve branches innervating the elbow joint were injected using the ultrasound-guided techniques developed in Phase I; and (2) blind injections (Group B), where the articular nerve branches were injected blindly using the anatomical landmarks determined in Phase I.

**Figure 1:**
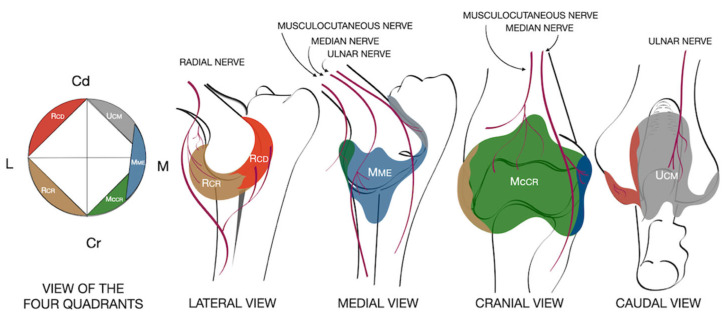
Schematic representation of the sensory innervation of the elbow. Areas are divided into cranial, caudomedial (gray), caudolateral (red), craniolateral (brown), cranial (green), and craniomedial (blue) quadrants. Craniolateral (Rcr) and caudolateral (Rcd) articular branches of the radial nerve; caudomedial articular branch of the ulnar nerve (Ucm); articular branch of the median nerve (Mme); and articular branch of the musculocutaneous nerve (Mccr). Dd: caudal; Cr: cranial; L: lateral; M: medial.

Each technique involved blind or ultrasound-guided injections at four injection points to target the articular branches of the nerves innervating the elbow joint [10]: two branches of the radial nerve, which supply the craniolateral (Rcr) and caudolateral (Rcd) quadrants of the joint capsule; the ulnar nerve, which supplies the caudomedial quadrant (Ucm); the median nerve, which supplies the medial quadrant (Mme); and the musculocutaneous nerve, which supplies the cranial quadrant (Mccr) (Figure 1). At each target point, 0.025 mL kg^–1^ of a dye solution was injected using a 22-gauge, 38 mm Quincke spinal needle (BD Spinal needle, Becton, Dickinson and Company, Franklin Lakes, NJ, USA). The dye solution was prepared fresh at the beginning of the study by mixing 1 mL of yellow permanent tissue colorant (Davison Marking System; Brandley Products Inc., Bloomington, MN, USA) with 250 mL of 0.9% NaCl solution (Baxter Healthcare Corporation, Deerfield, IL, USA).

### 2.1. Ultrasound-Guided PED Injection

**Figure 2:**
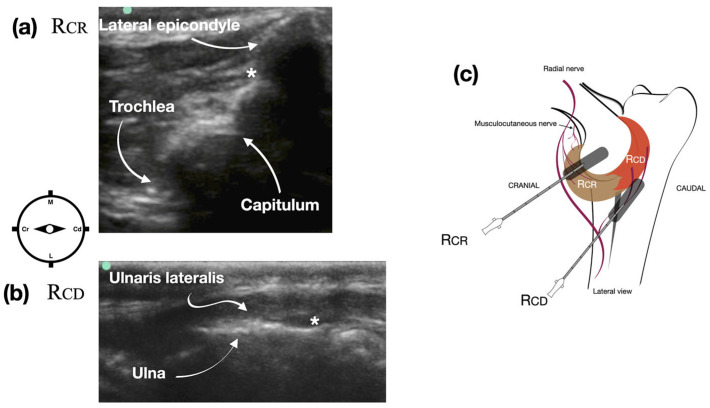
Ultrasound image of the pericapsular region of the elbow to target the articular branches of the radial nerve supplying the craniolateral quadrant of the joint capsule (Rcr) (**a**) and the caudolateral quadrant of the joint capsule (Rcd) (**b**). Schematic representation of the transducer placement and needle direction to target the articular branches of the radial nerve (**c**). * indicates the ultrasound target injection point. M: medial, L: lateral, Cr: cranial, Cd: caudal.

Based on the Phase I results, the injection site for accessing the articular branches of the radial nerve, which supply the craniolateral quadrant of the joint capsule (Rcr), was identified by positioning the ultrasound transducer perpendicular to the distal humerus (Figure 2). The transducer was gradually moved distally to visualize the lateral epicondyle, capitulum, and trochlea (Figure 2a). Once these anatomical landmarks were clearly defined, the needle was introduced from the cranial aspect of the distal humerus and advanced toward the craniomedial aspect of the lateral epicondyle. The dye was deposited in the subfascial plane between the extensor carpi radialis muscle and the periosteum of the bone (Figure 2a). To access the articular branches of the radial nerve, which supply the caudolateral quadrant of the joint capsule (Rcd), the transducer was positioned longitudinally along the olecranon on the caudolateral aspect of the elbow (Figure 2c). This positioning allowed visualization of the ulna and the ulnaris lateralis muscle. The needle was then introduced in a distal-to-proximal direction, and the dye was deposited in the subfascial plane between the ulnaris lateralis muscle and the periosteum of the ulna (Figure 2b).

**Figure 3:**
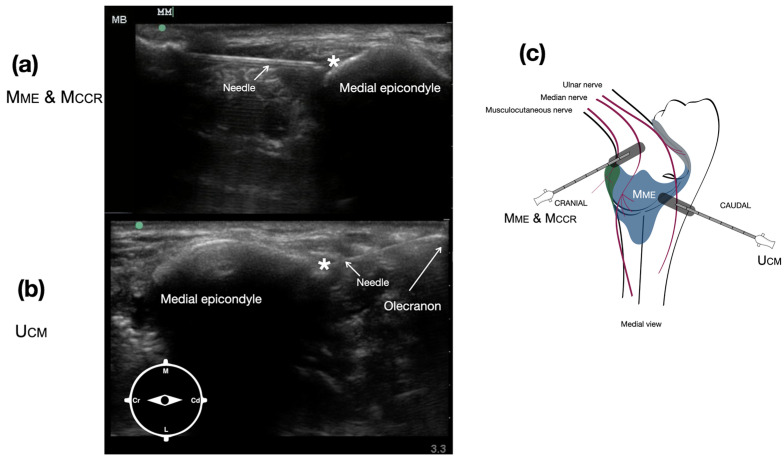
Ultrasound image of the pericapsular region of the elbow to target the articular branches of the median nerve (Mme) and the musculocutaneous nerve (Mccr) (**a**), which supply the medial and cranial quadrants of the joint capsule and ulnar nerve (Ucm) (**b**), supplying the caudomedial quadrant of the joint capsule. Schematic representation of the transducer placement and needle direction to target the articular branches of the musculocutaneous, median, and ulnar nerves (**c**). * indicates the ultrasound target injection point. M: medial, L: lateral, Cr: cranial, Cd: caudal.

The injection site targeting the articular branches of the median nerve (Mme) and the musculocutaneous nerve (Mccr), which supply the medial and cranial quadrants of the joint capsule, respectively, was identified as a common injection point. The transducer was positioned on the medial aspect of the brachial region, perpendicular to the long axis of the distal humerus, to visualize the belly of the biceps brachii muscle. The transducer was slid distally following the insertion tendon of the biceps until the medial epicondyle was visualized (Figure 3a). The needle was then introduced in a cranial-to-caudal direction, lateral to the belly of the biceps, and advanced until it contacted the craniolateral aspect of the medial epicondyle, where the dye was injected (Figure 3a).

To access the articular branches of the ulnar nerve (Ucm), which supply the caudomedial quadrant of the joint capsule, the transducer was placed on the caudomedial aspect of the elbow using the medial epicondyle and the olecranon as ultrasonographic landmarks (Figure 3b). The needle was introduced in a caudal-to-cranial direction and guided to the caudomedial aspect of the medial epicondyle, where the dye was deposited (Figure 3b).

### 2.2. Blind PED Injection

**Figure 4:**
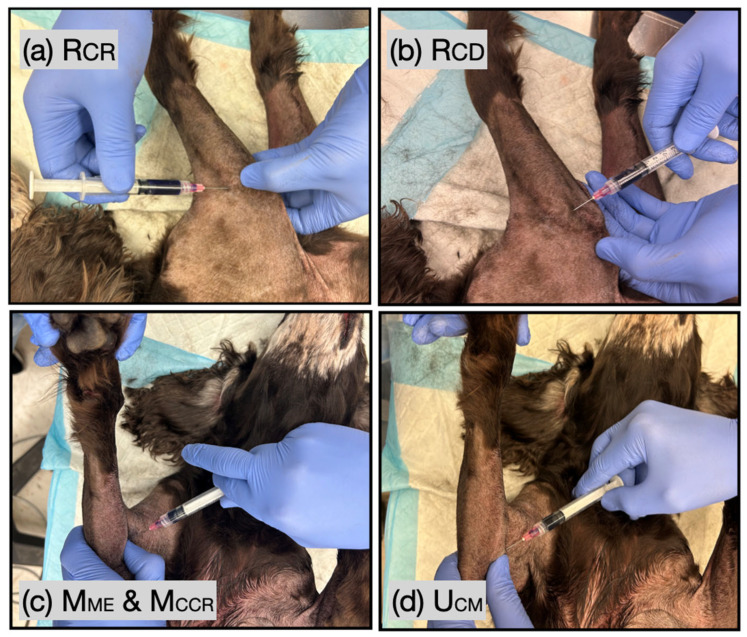
Injection points for blind injections targeting the articular branches of the radial nerve supplying the (**a**) craniolateral quadrant of the joint capsule (Rcr), the (**b**) caudolateral quadrant of the joint capsule (Rcd), the articular branches of the (**c**) median (Mme) and the musculocutaneous nerves (Mccr), and (**d**) the articular branches of the ulnar nerve (Ucm).

The blind PED technique was performed using anatomical landmarks to target the articular branches of the nerves innervating the elbow joint. For the Rcr injection, the craniomedial aspect of the lateral epicondyle was identified through palpation (Figure 4a). The needle was introduced from the cranial aspect of the distal humerus and directed toward the identified bony landmark. For the Rcd injection, a depression between the caudolateral and distal aspect of the lateral condyle and the ulna served as the anatomical landmark. The needle was inserted in a distal-to-proximal direction, targeting the flat lateral portion of the ulna, caudal to the proximal ends of the radius (Figure 4b).

A single injection site was used for the Mme and Mccr on the craniomedial aspect of the elbow. The belly of the biceps brachii muscle was palpated to locate its distal tendon of insertion. The needle was introduced in a cranial-to-caudal direction, lateral to the biceps tendon, and advanced until it contacted the craniolateral aspect of the medial epicondyle (Figure 4c).

The medial epicondyle and olecranon for the Ucm injection were palpated and used as anatomical landmarks. The needle was inserted in a caudal-to-cranial direction, targeting the caudomedial aspect of the medial epicondyle (Figure 4d). In all four injections, the dye was administered when the needle tip contacted the periosteum at each injection site. Before injection, negative pressure was applied in the syringe to rule out synovial liquid presence.

### 2.3. Nerve Stain Evaluation

After completing the blind and ultrasound-guided injections, the elbows were dissected as described in Phase I. Injections were considered successful if the dye solution stained the circumference of each articular nerve. For cases where articular nerve branches could not be identified during dissection, the successful injection was defined as staining in the region of the joint capsule associated with the quadrant innervated by the respective articular branches. The parent nerves originating all the articular branches were assessed for colorant stain.

The stained areas corresponding to each injection were excised en masse, preserved in 10% neutral buffered formalin, and submitted for histological analysis. For histological evaluation, the tissue samples were embedded in paraffin, sectioned at a thickness of 4 µm, and stained with hematoxylin and eosin. The histological analysis focused on determining the presence or absence of nervous tissue (nerves) within the samples.

### 2.4. Statistical Analysis

Considering an estimated success rate of 40% and 95% using blind and ultrasound-guided techniques, setting an alpha (probability of type I error) of 0.05 and a power of 0.8, a total of 20 elbows (10 canine cadavers) were deemed necessary to compare the effect of the two techniques. An interim statistical analysis was planned after 80% of the total study population (eight dogs). A sequential analysis with a maximum of two looks was performed with a Fisher’s combination test design, and a cumulative alpha spending of 0.04 on the main outcome was set to interrupt the study. The overall success rate (i.e., the total number of nerves stained) with each technique was compared between the two techniques using Fisher’s exact test. The area under the curve (AUC) of the successfully stained nerves was calculated considering the percentage of success in each elbow, and it was compared using an unpaired t-test. Continuous variables are shown as a median and interquartile range, while categorical data are shown as percentages. Statistical analyses were performed using GraphPad Prism Version 8.0 (GraphPad Software Inc., San Diego, CA, USA).

## 3. Results

- Phase I: Anatomical findings

**Figure 5:**
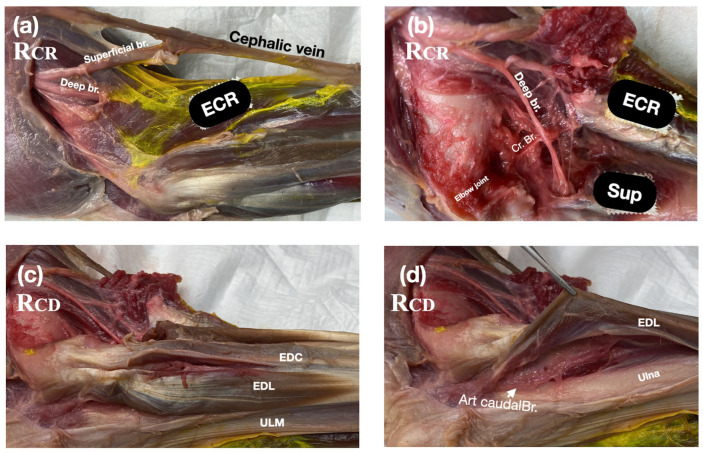
Anatomical dissection of the radial nerve showing the deep and superficial branches and areas of the craniolateral (Rcr) and caudolateral (Rcd) articular branches of the radial nerve. ECR: extensor carpi radialis muscle; EDC: extensor digitorum communis muscle; EDL: extensor digitorum lateralis muscle; Sup: supinator muscle; ULM: ulnaris lateralis muscle.

The initial anatomical dissections revealed that the radial nerve was located beneath the deep brachial fascia, between the triceps and brachialis muscles. At this point, the radial nerve bifurcated into two branches, a superficial branch that coursed along the cephalic vein and a deep branch that ran distally toward the extensor carpi radialis muscle (Figure 5a). The deep branch of the nerve passed beneath the extensor carpi radialis before sending a cranial articular branch to the craniolateral aspect of the elbow joint capsule (Rcr) (Figure 5b). As the deep branch continued distally, it ran deep to the supinator muscle, becoming dorsal to the supinator while lying beneath the extensor carpi radialis, ultimately positioning itself on the dorsolateral aspect of the radius sending the Rcd branch to the joint capsule.

**Figure 6:**
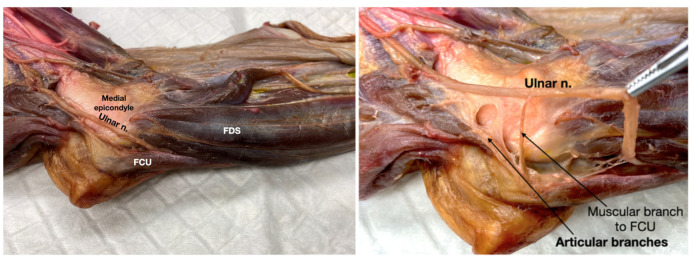
Anatomical dissection of a left thoracic limb of a dog (medial view) showing the ulnar nerve with its muscular and articular branches. FCU: flexor carpi ulnaris muscle; FDS: flexor digitorum superficialis muscle.

The ulnar nerve contributed a single articular branch to the caudomedial aspect of the elbow joint capsule (i.e., the Ucm). Dissections on the mediocaudal aspect of the distal humerus showed that the ulnar nerve runs under the antebrachial and deep fascia, located caudal to the medial condyle and deep to the flexor carpi ulnaris (FCU), and caudal to the flexor digitorum superficialis (FDS), resting upon the deep digital flexor (DDF) (Figure 6). A muscular branch to the FCU was observed originating at the level of a line connecting the medial condyle with the proximal part of the olecranon. The articular branch (i.e., Ucm) arose proximally to this muscular branch, embedded within dense connective tissue (Figure 6.

The median nerve was also found to provide a small articular branch to the medial aspect of the elbow joint capsule (i.e., Mme). Located cranially to the ulnar nerve in the distal part of the brachial region, the median nerve was associated closely with the brachial artery and vein. As it crossed the flexor surface of the elbow joint cranial to the medial epicondyle, it traveled distally, caudal to the biceps brachii muscle and adjacent to the transverse cubital artery. The median nerve passed deep to the pronator teres muscle, entering the caudal group of flexor muscles within the antebrachium. Under the pronator teres muscle, the nerve gave rise to a small articular branch (i.e., the Mme), which passed superficially to the medial collateral ligament of the elbow. The musculocutaneous nerve was found to send a small branch to the craniolateral part of the elbow joint capsule (i.e., the Mccr) when the nerve crosses the cranial surface of the elbow joint, between the biceps brachii and brachialis muscles.

Ultrasonography and superficial bony landmarks allowed the identification of four injection points to target the articular nerves innervating the craniolateral (Rcr), caudolateral (Rcd), craniomedial (Mccr), medial (Mme), and caudomedial (Ucm) aspects of the elbow joint capsule.

- Phase II: Comparison of ultrasound-guided and blind PED techniques

The interim statistical analysis revealed significant differences in the overall success rates between the ultrasound-guided and blind techniques using eight canine cadavers. Therefore, only 16 thoracic limbs obtained from eight canine cadavers weighing 11 (7.8–23.8) kg were used.

The ultrasound-guided technique achieved a significantly higher overall success rate of 77.5% compared to 45% for the blind technique (*p* = 0.005). As measured by the AUC, the total nerves stained was significantly higher for the ultrasound-guided technique (540 ± 75.8) than for the blind technique (320 ± 92.2, *p* = 0.0001).

**Figure 7:**
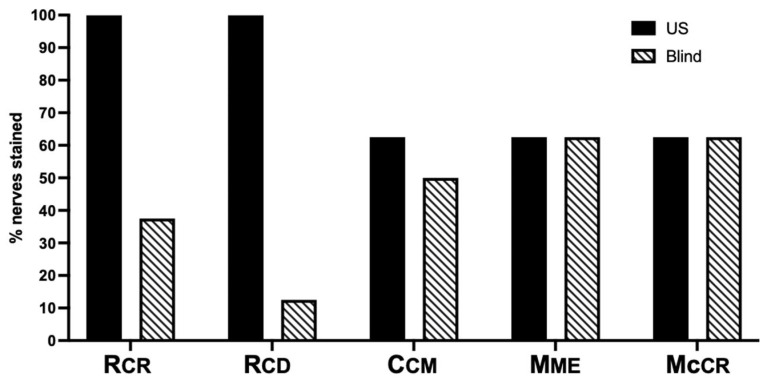
Percentage of success of each of the articular branches stained using the ultrasound-guided (US) and the blind techniques. Craniolateral (Rcr) and caudolateral (Rcd) articular branches of the radial nerve; caudomedial articular branch of the ulnar nerve (Ucm); articular branch of the median nerve (Mme); and articular branch of the musculocutaneous nerve (Mccr).

The ultrasound-guided technique successfully stained the Rcr and Rcd branches in 100% of cases, while the blind technique achieved only 37% and 12.5% for the Rcr (*p* = 0.02) and Rcd (*p* = 0.001) branches, respectively (Figure 7). The success rate of staining the Ucm was 62.5% and 50% for the ultrasound-guided and blind techniques, respectively (*p* > 0.99). Both the ultrasound-guided and blind techniques stained the Mme and the Mccr branches in 62.5% of the injections (*p* > 0.99) (Figure 7). In all unsuccessful injections, the dye solution was found to spread within muscles or within connective tissues unrelated to the target site.

A total of seven out of 32 (21.9%) parent nerves were stained in each group. Specifically, the Rcr injection stained two radial nerves in each group. The Ucm injection stained three ulnar nerves in the US group and two ulnar nerves in the blind technique group. The Mme injections stained one median nerve in the US group and three median nerves in the B group. Moreover, the Mccr injection stained one musculocutaneous nerve in one case from the US group. The presence of intracapsular dye solution was identified in three cases (i.e., two in the US group and one in the B group).

**Figure 8:**
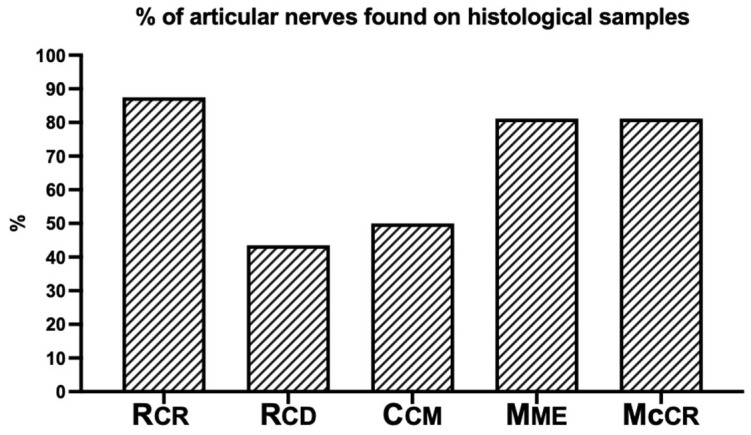
Percentage of articular nerves found in the histological samples in both the ultrasound-guided and blind groups. Craniolateral (Rcr) and caudolateral (Rcd) articular branches of the radial nerve; caudomedial articular branch of the ulnar nerve (Ucm); articular branch of the median nerve (Mme); and articular branch of the musculocutaneous nerve (Mccr).

Articular nerves were found in 70% and 69.2% of the histological samples for the US and B groups, respectively (*p* > 0.99). However, the histological analysis found that identifying the articular nerves remained inconsistent (Figure 8).

## 4. Discussion

The present study demonstrated that articular nerves innervating the elbow joint capsule in dogs could be targeted using a four-quadrant pericapsular injection technique. The results indicated the superiority of the ultrasound-guided PED technique over the blind approach, achieving a significantly higher success rate in staining the target nerves, particularly the articular branches of the radial nerve.

The ultrasound-guided approach consistently stained the articular branches of the radial nerve (i.e., Rcr and Rcd) in all cases. However, the success rate for staining the articular branches originating from the ulnar (Ucm), median (Mme), and musculocutaneous (Mccr) nerves was 62.5%, underscoring the need for further refinement of the PED technique.

Regional anesthesia is increasingly utilized in veterinary practice due to its benefits in managing perioperative [12] and chronic pain [7,13]. Various techniques have been developed to target brachial plexus components, providing adequate analgesia [14,15,16]. However, these methods often result in varying degrees of motor block, limiting their suitability for long-term pain management. Recently, the pericapsular hip desensitization (PHD) technique has been introduced as a promising approach to block the articular branches of the hip joint in dogs without inducing motor impairment [7]. Based on the principles of the PHD technique, this study proposed a similar approach called the PED technique, which targets specific anatomical points around the elbow joint capsule to desensitize its articular branches.

In human medicine, radiofrequency ablation has been recognized as an effective method for pain relief and reducing opioid use, particularly in postoperative or inoperable lower extremity joint pain cases [17,18]. Similarly, thermal radiofrequency ablation of the saphenous nerve has been successfully used to treat naturally occurring stifle osteoarthritis in dogs [19]. However, the application of peripheral nerve block or radiofrequency ablation techniques for managing osteoarthritic pain in the elbow joint remains limited. The current human-related literature highlights a lack of procedural development in this area, particularly for the upper extremities [20]. A primary challenge lies in the insufficient knowledge regarding the precise location of the articular branches innervating the elbow joint and their spatial relationships with ultrasonographic landmarks.

Chronic elbow pain, particularly that associated with osteoarthritis, presents a significant clinical challenge due to the limitations and potential complications of existing treatment modalities, including both conservative management and surgical intervention [21]. While ultrasound-guided peripheral nerve block and radiofrequency ablation have shown efficacy in managing chronic pain in other joints, their application in the elbow has been hindered by the lack of detailed anatomical knowledge regarding the articular innervation of the joint. Recently, a detailed anatomical study conducted in embalmed human cadavers addressed this gap by providing a comprehensive three-dimensional (3D) anatomical characterization of the articular branches innervating the elbow joint capsule [22]. Similar to our findings, the authors of this human study identified specific bony and soft tissue landmarks that could facilitate the ultrasound-based localization of these articular branches.

In veterinary medicine, anatomical studies addressing the innervation of the elbow joint capsule in dogs remain limited [9,10]. Zamprogno et al. [10] reported that the elbow joint receives articular branches from the radial, ulnar, median, and musculocutaneous nerves in dogs. However, a recent study highlighted variability in the number and location of these branches, particularly from the median and radial nerves, among individual dogs [9]. Consistent with the findings of the present study, some specimens lacked identifiable nerve contributions, potentially due to anatomical variations or dissection limitations [22]. The small size of these nerves further complicated their identification in the dissected tissues.

An advanced method for identifying the nerve supply to the elbow was employed in a human cadaveric study by Arnold et al. [22]. A 3D innervation model was developed through a combination of dissection, digitization, and reconstruction. Articular branches were exposed in cadaveric specimens, and their three-dimensional coordinates were recorded using a Microscribe digitizer, with reference screws placed in bony landmarks to ensure accuracy. Following skeletonization and laser scanning, the data were imported into professional 3D computer graphics software to create interactive models. Custom plug-ins enabled precise 3D reconstruction, allowing detailed visualization of nerve pathways and anatomical relationships. This technology may also be applied in the future to better characterize the complex sensory innervation of the elbow joint in dogs.

One of the primary objectives of interventional pain procedures is to target sensory nerves while avoiding motor nerve compromise. This study evaluated the impact of the PED technique on parent nerves, finding that approximately 21.9% of parent nerves were stained independently of the technique used. Reducing the injectate volume may help minimize parent nerve involvement in clinical cases. However, a further reduction in the used volume could potentially reduce the success rate of the technique in reaching the target articular nerves. In three cases, another observed complication was dye solution inside the joint capsule, likely caused by direct intracapsular injection. These events highlight the potential risk when performing invasive procedures such as radiofrequency ablation or neurolytic injections. Further refinements of the PED technique are necessary to minimize these potential complications.

Two different dye solutions were used for the different phases of the study. The decision to switch from new methylene blue to the tissue marker solution was based on the tendency of methylene blue to diffuse extensively through tissues [23,24,25]. As previously reported, this high diffusibility can compromise the precision of evaluating injection accuracy [26]. Methylene blue can spread through intact tissues, potentially staining target nerves during dissection even when the injection was placed incorrectly. In contrast, the tissue marker solution remains confined to the injected fascial plane, reducing the likelihood of nerve staining when the needle is not accurately positioned.

Several limitations of this study should be noted. First, the study was conducted on canine cadavers, where anatomical structures (e.g., vessels, muscles, and ligaments) may differ in appearance on ultrasonography compared to live animals. This could affect the execution and accuracy of the PED technique in clinical settings. Additionally, the spread of the injectate in cadavers may not replicate its behavior in live animals, necessitating cautious interpretation of the results. In some cases, visualizing ultrasonographic landmarks proved challenging, especially in small dogs, where the limited contact area between the ultrasound transducer and the skin impeded imaging clarity. In several instances, target nerves could not be located during gross anatomical evaluations, leading to success being defined as staining the area expected to contain the articular branch rather than direct nerve staining. Moreover, in some histological specimens, nerves were not identified despite performing multiple-step sections (i.e., recuts) through entire paraffin blocks containing the samples. This may have been due to the absence of the target nerve in the submitted tissue or failure to capture these small nerves in the histological sections. An additional limitation of this study is the small injection volume (0.025 mL kg^–1^) used. While this volume was chosen to enhance precision within the small anatomical target areas, we acknowledge that it may increase the likelihood of injection failure. Conversely, larger volumes carry a greater risk of diffusion to the parent nerves, which could limit the clinical application of neurolytic agents. Finally, although the limbs were examined for gross anatomical abnormalities and only those from dogs with apparently normal elbows were included, diagnostic imaging (e.g., computed tomography or radiography) was not performed prior to enrollment. Therefore, the presence of underlying joint abnormalities in the selected limbs could not be definitively ruled out. These challenges emphasize the need for further refinement of techniques, methodologies, and injectate volumes to improve consistency and accuracy in identifying and targeting articular nerves.

## 5. Conclusions

In conclusion, this study lays the groundwork for advanced anatomical and clinical research on the PED technique in live animals, particularly its potential for long-term pain management in dogs with elbow osteoarthritis. By targeting sensory branches, the PED technique may offer effective analgesia and enhance the quality of life for affected canine patients.

## Data Availability

The raw data supporting the conclusions of this article will be made available by the authors on request.
